# Artificial intelligence in eye care: a cautious welcome

**Published:** 2022-06-07

**Authors:** Andrew Bastawrous, Charles Cleland

**Affiliations:** 1 Co-Founder & CEO: Peek Vision Ltd and Professor in Global Eye Health: London School of Hygiene and Tropical Medicine, London, UK.; 2 Clinical Research Fellow: International Centre for Eye Health, London School of Hygiene & Tropical Medicine. London, UK.

**Artificial intelligence is being positioned as a technology that can transform health care. However, there are pitfalls.**

**Figure:**
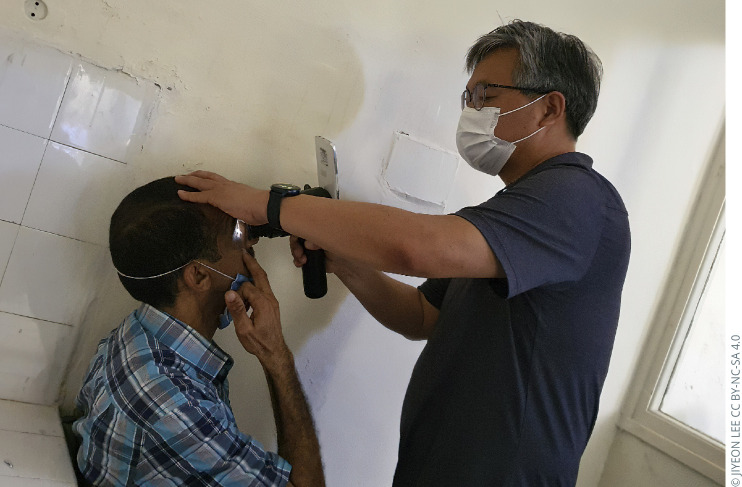
A portable fundus camera equipped with AI software is used to screen for diabetic retinopathy. **MOROCCO**

Artificial Intelligence (AI) is a field that aims to develop computers with the same capabilities as humans, such as the ability to perceive, learn, problem solve, and manipulate.

One of the most well-known early applications of AI is the development of chess computers, culminating in the ‘Deep Blue’ computer which beat the world's leading chess player at that time, Gary Kasparov, in 1997. However, this was a very narrow and specific use for AI. It would take a rapid rise in computing power over the next two decades, plus the availability of vast sets of data in the areas of health, economics, and demographics (to name a few), before AI started to make a difference in health care – with ophthalmology as one of its first applications.

The key benefits of AI within health care are improved accuracy, decision making, and efficiency, which can relieve humans of mundane, repetitive tasks, thereby maximising their unique value within the workforce. The first AI-based medical device to get Food and Drug Administration (FDA) approval in the United States was a tool for automatically grading retinal images, known as IDx. Several retinal grading systems now exist, and they are much faster than human graders, thereby reducing the cost, burden, and delays associated with human grading.^1^

The use of AI for medical image analysis involves machine learning: ‘training’ the AI system using large data sets, such as sets of retinal images with features labelled by experts for the machine to ‘learn’ from. Provided that the machine has learnt from a large enough number of labelled images, it can then grade images it has never ‘seen’ before.

AI is being positioned as a technology that can bridge the health inequity gap. However, there are pitfalls, as stated in the *Lancet Global Health* Commission on Global Eye Health: “Technological developments such as …. artificial intelligence offer the potential to revolutionise eye health care in the next decade by delivering affordable, high-quality services to remote areas. However, caution is needed to ensure all populations benefit from these developments.”^2^

The accuracy of an AI system depends on the quality and suitability of the data it is trained on. In DR screening, the need for large, well-managed databases of retinal images for the AI to learn from leads to a bias towards developing AI systems that are trained to identify diabetic retinopathy (DR) in populations for whom such datasets already exist, typically in well-resourced health systems. This further leaves behind those living in low-resourced health settings (an issue which has recently become known as ‘health data poverty’).^3^

It is not immediately obvious whether AI systems trained on high-income datasets will be able to achieve the same levels of accuracy in populations elsewhere, and they must therefore be tested locally – and evaluated against skilled human graders – before being implemented.

Eye health represents one of the most exciting areas in medicine where AI is likely to have a large impact. However, the hard and complex work of integration into health services has yet to be realised. Activities that enable task shifting and more affordable, local services, such as DR grading at primary health facilities, are needed to ensure better access and lower the pressure on the health service.

Even if the potential of AI for DR grading is fully realised, the 4.4 million people estimated to have any level of vision impairment from diabetic retinopathy equates to a small fraction of the 1.1 billion people living with vision impairment globally.^2^ Leading causes of vision loss, such as untreated cataract and refractive error, have yet to receive the same level of interest and investment as DR.

If we are truly to realise the potential of AI in health care and eye care, a more equitable and purposeful design of technologies and incentive systems is required.
